# Nitrogen Self-Doped Activated Carbons Derived from Bamboo Shoots as Adsorbent for Methylene Blue Adsorption

**DOI:** 10.3390/molecules24163012

**Published:** 2019-08-20

**Authors:** Bingbing Mi, Jingxin Wang, Hongzhong Xiang, Fang Liang, Jianfei Yang, Zixing Feng, Tao Zhang, Wanhe Hu, Xianmiao Liu, Zhijia Liu, Benhua Fei

**Affiliations:** 1 International Centre for Bamboo and Rattan, Beijing 100102, China; 2 School of Natural Resources, West Virginia University, Morgantown, WV 26506, USA

**Keywords:** bamboo shoots, nitrogen self-doped, activated carbon, methylene blue, adsorption

## Abstract

Bamboo shoots, a promising renewable biomass, mainly consist of carbohydrates and other nitrogen-related compounds, such as proteins, amino acids and nucleotides. In this work, nitrogen self-doped activated carbons derived from bamboo shoots were prepared via a simultaneous carbonization and activation process. The adsorption properties of the prepared samples were evaluated by removing methylene blue from waste water. The factors that affect the adsorption process were examined, including initial concentration, contact time and pH of methylene blue solution. The resulting that BSNC-800-4 performed better in methylene blue removal from waste water, due to its high specific surface area (2270.9 m^2^ g^−1^), proper pore size (2.19 nm) and relatively high nitrogen content (1.06%). Its equilibrium data were well fitted to Langmuir isotherm model with a maximum monolayer adsorption capacity of 458 mg g^−1^ and a removal efficiency of 91.7% at methylene blue concentration of 500 mg L^−1^. The pseudo-second-order kinetic model could be used to accurately estimate the carbon material’s (BSNC-800-4) adsorption process. The adsorption mechanism between methylene blue solution and BSNC-800-4 was controlled by film diffusion. This study provides an alternative way to develop nitrogen self-doped activated carbons to better meet the needs of the adsorption applications.

## 1. Introduction

Water pollution and global climate change are major environmental concerns due to the production and use of fossil fuels. It is well known that waste water often contains various types of synthetic dyes [1], each dye is harmful to the health of humans or animals. For instance, methylene blue (MB), as one of the important synthetic dyes, is widely used in chemistry, biology, medical science and dyeing industries [2]. The large doses of MB (>7.0 mg kg^−1^) can cause high blood pressure, mental disorder, nausea, abdominal pain [3]. Therefore, it is necessary to remove the hazardous dyes before waste water is discharged or used. There are many ways to remove MB from waste water, such as adsorption [4], coagulation and flocculation [5], oxidation [6], photocatalytic [7], membrane [8] and ion exchange [9]. Among those, adsorption using activated carbon is considered as one of the most effective and simple methods. Meanwhile, the efforts to remove pollutants from waste water require the use of functional materials, while porous carbon materials exhibit a superior performance and have been widely used in water purification, due to their moderate pore size, pore volume, surface area and functional groups. To reduce the cost of raw materials and the preparation of carbon material, it is important to find a cheaper precursor and a more facile synthetic approach. Many studies have reported on the development of activated carbons from biomass, such as bamboo [10,11], tea waste [12], reed [13], seaweed [14] and banana peel [15]. The advantage of using biomass to prepare porous carbon materials is that the raw materials are abundant, cheap, renewable, and exhibit promising application as adsorbents in filtrating and purifying waste water [16].

Recently, nitrogen-doped carbon materials derived from biomass have been attracted more attention in the application of adsorption due to their superior properties. The existence of nitrogen can increase the number of basic sites, improve the capacitance performance on the surface of the carbons and increase the wettability between the carbon surface and aqueous solution [17,18,19]. Tan et al. [20] prepared carbon nitrides by refluxing under nitrogen and used them as adsorbents in the adsorption of MB molecules and heavy metal ions Pb^2+^ (720 mg g^−1^), Cd^2+^ (480 mg g^−1^) and As (V) (220 mg g^−1^). They found that the obtained carbon nitrides had a high performance for water treatment. Zhou et al. [21] reported that hierarchical nitrogen-doped porous carbons derived from biomass had a promising adsorption capacity for MB (1551 mg g^−1^). All the mentioned above studies estimated that nitrogen-doped carbon materials have a large potential in the field of adsorption. 

For adsorption, surface area, pore volume, nitrogen content and other factors have significant effect on the connection between the adsorbent and the adsorbate. Thus, it is necessary to design and prepare large surface area, controllable pore structures, high nitrogen content, low cost and environmentally friendly nitrogen-doped porous carbon materials derived from biomass. As one of the renewable biomass resources, bamboo shoots have abundant carbohydrate, amino acids and nucleotides except for cellulose, hemicelluloses and lignin [22], which can be used as nitrogen and carbon resources for the preparation of nitrogen-doped carbon materials. In addition, it is less expensive for bamboo shoot as carbon precursor than other materials [23,24,25,26]. To the best of our knowledge, there is a lack of sufficient information on porous nitrogen self-doped activated carbon materials from bamboo shoots to remove MB of waste water. 

Thus, this study aimed to synthesize the hierarchically porous nitrogen self-doped activated carbon materials from bamboo shoots (BSNCs) via simultaneous carbonization and activation. We investigated the adsorption properties of porous nitrogen-doped activated carbons from bamboo shoots for the removal of MB, specifically the factors affecting the adsorption of MB, including contact time, initial dye concentration and solution pH. Langmuir, Freundlich and Temkin of isotherm models, the pseudo-first-order and pseudo-second-order of kinetic models were also used to understand the adsorption mechanism of MB using the BSNCs. 

## 2. Results and Discussion

### 2.1. Characterization of BSNCs

**Figure 1:**
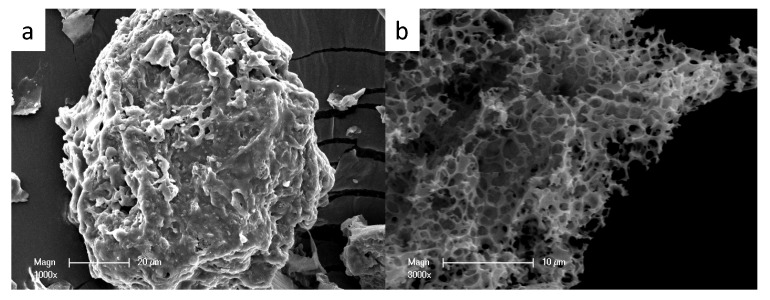
SEM images of (**a**) BS-800 and (**b**) BSNC-800-4.

Figure 1 shows the morphology of BSNC-800-4 (a present SEM image as the BSNCs), with BS-800 as a reference. There were a number of pores for BSNC-800-4 (Figure 1b), and part of them were collapsed, due to the interaction of the substances, such as K_2_CO_3_, H_2_O, CO_2_, in which KHCO_3_ was decomposed at elevated temperature. As a comparison, BS-800 (Figure 1a), which was directly carbonized at 800 °C in nitrogen atmosphere, showed irregular surface with few pores. 

**Figure 2:**
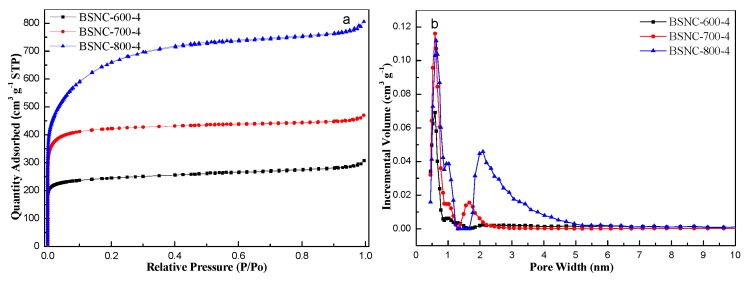
(**a**) Nitrogen adsorption/desorption isotherms and (**b**) pore size distribution of BSNC-600-4, BSNC-700-4 and BSNC-800-4.

To further study the porous structure of BSNCs, N_2_ sorption isotherms were carried out at 77 K. As shown in Figure 2, the isotherm of BSNCs was type I according to IUPAC classification, suggesting the existence of different pore sizes varied from micro- to macro-pore. The steep increase at low relative pressure (*P/P*_0_ < 0.1) indicated the presence of micropores. At relative pressure of 0.1–0.9 revealed the existence of mesopores, and the tails at a relative pressure of 1.0 indicated the existence of macropores. This proved that BSNCs had hierarchical pore structures. Table 1 shows the porosity properties and chemical composition of BSNCs. Compared to the specific surface area of BS-800 (12.2 m^2^ g^−1^), BSNCs had large specific surface area and BSNC-800-4 had the largest one (2270.9 m^2^ g^−1^) among them. And the total pore volume of BSNC-800-4 was 1.25 m^3^ g^−1^. The average pore size of BSNC-800-4 was 2.19 nm, which was also higher than that of BSNC-600-4 and BSNC-700-4. High specific surface area and pore size could be useful for adsorption of MB.

**Figure 3:**
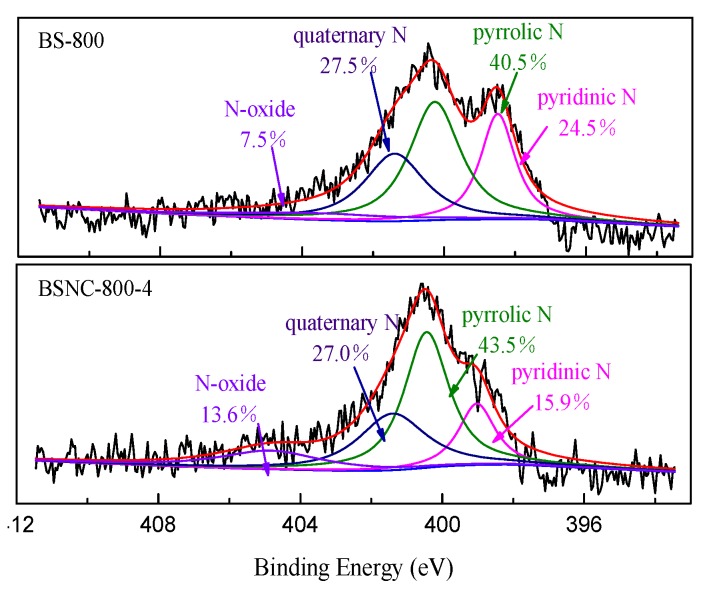
XPS spectra of N1s for BS-800 and BSNC-800-4.

The elemental analysis showed that carbon, hydrogen, nitrogen and oxygen co-existed in the obtained samples (Table 1). BS-800 had a nitrogen content of 4.64%, indicating that it obtained a high nitrogen content without adding nitrogen source during synthesizing process. BSNC-800-4 still had a nitrogen content of 1.06% even though the calcination temperature caused the degradation of the nitrogen functional groups [27,28]. According to XPS spectra of N1s (Figure 3), there were four different types of nitrogen on BS-800 and BSNC-800-4, including pyridinic N (398.5 eV), pyrrolic N (400.3 eV), quaternary N (401.3 eV) and N-oxide (404.8 eV). Pyrrolic N and quaternary N were two main types of N. After calcination, the amounts of pyrrolic N and N-oxide increased, while the amount of pyridinic N and quaternary N decreased. This phenomenon was due to the rearrangement in C-N bond during synthesizing process of BSNCs. Wang et al. [29] found that the N function groups on the surface of the carbon materials played a vital role in the adsorption process except for surface area and pore structures. Tan et al. [21] reported that the presentence of pyrrolic N and pyridinic N had an effect on the adsorption of heavy metal ions Pb^2+^, Cd^2+^ and As (V). Based on the above analysis, the properties of the samples we obtained, including porosity, chemical composition, functional groups would have effects on the adsorption capacity and removal efficiency of MB. 

### 2.2. Adsorption Properties of BSNCs

#### 2.2.1. Effect of Preparation Process of BSNCs on Its Adsorption Properties

**Figure 4:**
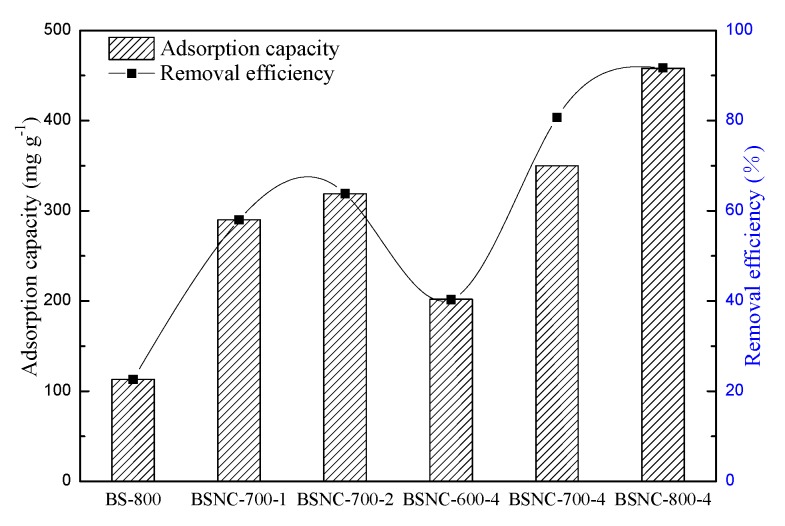
The adsorption capacity and removal efficiency of BSNCs; left y-axis represents the adsorption capacity, right y-axis represents the removal efficiency.

To investigate effect of the preparation process of BSNCs on its adsorption properties, 50 mg of BSNCs was added to the MB concentration of 500 mg L^−1^ at temperature of 298 K with contact time of 360 min. The results are shown in Figure 4. BS-800 had the lowest adsorption capacity and removal efficiency with the value of 113.2 mg g^−1^ and 22.6%. BSNC-800-4 had the highest adsorption capacity and removal efficiency with the value of 458 mg g^−1^ and 91.7%. While the mass ratio was 4, the adsorption capacity of BSNC-600-4, BSNC-700-4 and BSNC-800-4 gradually increased from 202 to 458 mg L^−1^. Similarly, when the calcined temperature was 700 °C, the adsorption capacity of BSNC-700-1, BSNC-700-2 and BSNC-700-4 also gradually increased from 290 to 350 mg L^−1^. This indicated that the synthesizing process including calcination temperature and mass ratio of bamboo shoot particles and KHCO_3_ improved the adsorption capacity and removal efficiency of BSNCs. 

#### 2.2.2. Effect of pH

**Figure 5:**
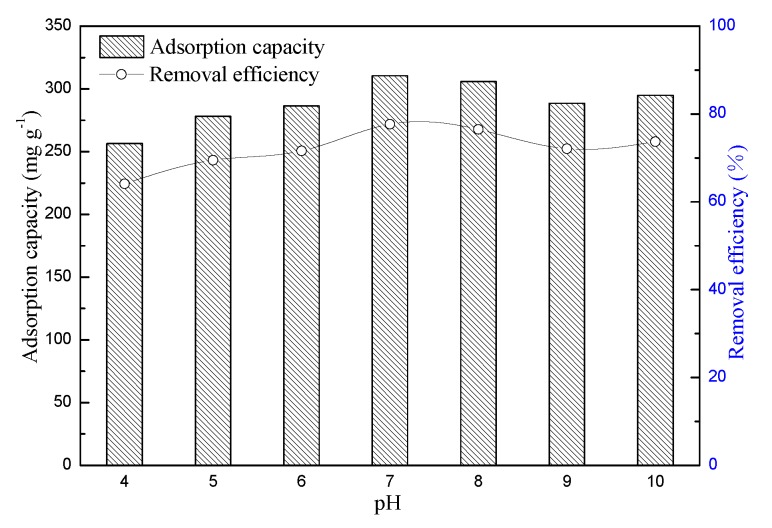
Effect of pH on the adsorption capacity and removal efficiency of BSNC-800-4; left y-axis represents the adsorption capacity, right y-axis represents the removal efficiency.

The adsorption capacity and removal efficiency of MB for BSNC-800-4 were examined with a pH of 4 to 10. A dosage of 30 mg, initial MB concentration of 500 mg L^−1^, temperature of 298 K, 200 rpm and contact time of 360 min. The initial pH of MB solution was adjusted by 0.1 mol L^−1^ NaOH or HCl separately. Figure 5 showed the adsorption capacity and removal efficiency increased when the pH value increased from 4 to 7. Then it had a slight decrease with the increase of pH value. This confirmed that the pH value of dye solution affected the adsorption capacity and removal efficiency of BSNC-800-4, and is similar to the results of previous studies [30,31]. It was also indicated that the removal efficiency was lower under acid conditions. This might be due to the presence of excess of H^+^ ions which competed adsorption sites with MB or due to the force between BSNC-800-4 and MB solution. When pH value was higher than 7, the surface of BSNC-800-4 may become negatively charged, which favors the adsorption of MB cations due to the formation of an electric double layer changes its polarity. The pH value should be adjusted to 7 while using BSNC-800-4 to remove MB from waste water. It also exhibited that the adsorption capacity of BSNC-800-4 is not clearly affected by the initial concentrations of MB. 

#### 2.2.3. Effect of Initial Concentration of MB and Contact Time

**Figure 6:**
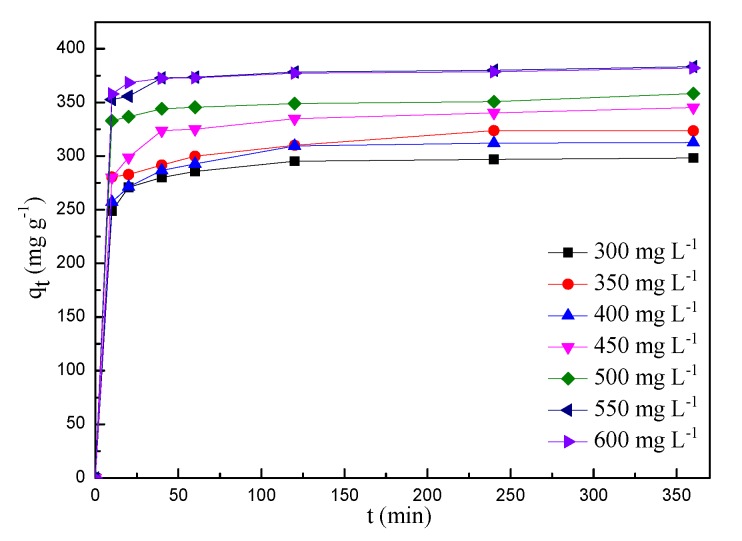
Adsorption capacity vs. contact time at different MB concentrations on BSNC-800-4.

**Figure 7:**
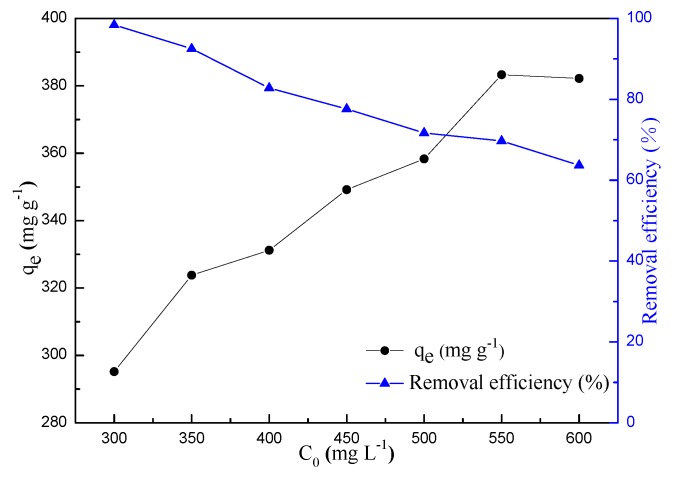
Adsorption capacity and removal efficiency vs. initial MB concentrations on BSNC-800-4; left y-axis represents the adsorption capacity, right y-axis represents the removal efficiency.

To investigate the effect of initial MB concentrations and contact time on adsorption property and removal efficiency of BSNC-800-4, seven different initial MB concentrations (300–600 mg L^−1^) were chosen and sampled at different intervals (10–360 min). As shown in Figure 6. The adsorption capacity increased of MB concentrations and reached to the dynamic equilibrium, mainly attributable to its high surface area, large pore volume, as well as the functional groups, such as the types of N and the content of O on its surface. It is clear that the adsorption curves of MB using BSNC-800-4 presented two steps, including a fast adsorption and the adsorption equilibrium period. The adsorption capacity of BSNC-800-4 quickly increased up to an equilibrium state in 60 min. There was little difference of the adsorption capacity between the MB concentration of 550 mg L^−1^ and 600 mg L^−1^. This indicated that while the dosage of BSNC-800-4 was constant, there was not sufficient sorption sites on its surface to attach the MB molecules at higher initial dye concentration. With an increase of MB concentrations from 300 to 600 mg L^−1^, the adsorption capacity and the removal efficiency up to equilibrium varied from 295.2 mg g^−1^ (98.4%) to 382.3 mg g^−1^ (63.7%) (Figure 7), due to the concentration of MB could provide the necessary driving force to overcome the mass transfer resistance between aqueous and BSNC-800-4. BSNC-800-4 had the superior adsorption capacity and removal efficiency at 550 mg L^−1^ of the MB concentration. A similar phenomenon was also found for adsorption of methylene dye on tea waste [12] and banana peel [15].

### 2.3. Adsorption Isotherms

Langmuir, Freundlich and Temkin isotherms were used to described the adsorption equilibrium of MB as the conventional adsorption isotherm models did [32]. Langmuir isotherm model [33] is monolayer adsorption onto a surface with the binding sites, is given as:

(1) $$\frac{C_{e}}{q_{e}}=\frac{1}{K_{L}q_{m}}+\frac{C_{e}}{q_{m}}$$

Freundlich isotherm model [34] is a multilayer adsorption on a heterogeneous surface and the heat of adsorption is not uniform between the molecules. The Equation (2) of Freundlich isotherm model is expressed as follows:

(2) $$lnq_{e}=lnK_{F}+\frac{1}{n}lnC_{e},$$

where *C_e_* is the equilibrium concentrations of MB (mg L^−1^), *q_m_* is the maximum adsorption capacity (mg g^−1^), *K_L_* is the Langmuir constant related to the rate of adsorption (L mg^−1^), which can be calculated from the plot *C_e_/q_e_* vs. *C_e_*. *K_F_* is the Freundlich constant and 1/n is the heterogeneity factor, which can be obtained from the plot *lnq_e_* vs. *lnC_e_*.

Temkin isotherm model [35] is denoted by Equations (3) and (4):

(3) $$q_{e}=BlnK_{T}+BlnCe$$

(4) $$B=\frac{RT}{b}$$

where *q_e_* is the adsorption capacity at equilibrium (mg g^−1^), *C_e_* is the equilibrium concentrations of MB (mg L^−1^). *R* is the general gas constant (8.314 J mol^−1^ K^−1^), *T* is absolute temperature (K), *K_T_* is the equilibrium binding constant, which can be calculated from the plot *q_e_* vs. *lnC_e_* (L mg^−1^).

**Figure 8:**
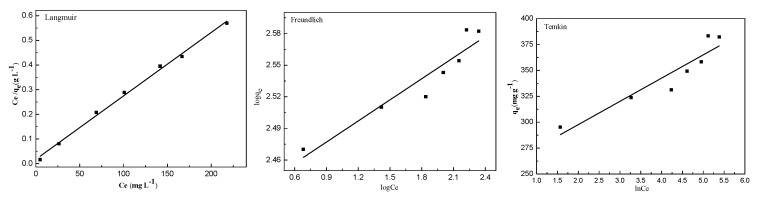
Linear regression of MB on BSNC-800-4 at 298 K with Langmuir isotherm model, Freundlich isotherm model and Temkin isotherm model.

Adsorption isotherms can be used to describe the interaction between the adsorbate and the carbonaceous adsorbent. In this study, there had a comparison of liner among Langmuir, Freundlich and Temkin isotherm models (Figure 8, Table 2). It was found that R^2^ of Langmuir isotherm was higher than that of other models, indicating that the calculated adsorption capacity from Langmuir isotherm presented the experimental data better. The results also indicated that the adsorption of MB molecules on BSNC-800-4 surfaces were mainly monolayer adsorption. And the maximum of monolayer adsorption capacity of MB was 384.6 mg g^−1^ at 298 K. The adsorption capacity of MB for BSNC-800-4 was better than that of other biomass, such as magnetic porous carbon fibers (143.0 mg g^−1^) [36], swede rape straw (246.4 mg g^−1^) [37], *Catalpa bignonioides* (271.0 mg g^−1^) [38], *Cortaderia selloana* flower spikes (66.2 mg g^−1^) [39]. The high MB adsorption capacity could be related to its hierarchical porous structures and the presence of functional groups (e.g., nitrogen groups and basic groups) on the surface of BSNC-800-4. Therefore, the functional groups and the porous properties on the surface of the carbon materials should be considered when used as the adsorbents for adsorption.

In addition, the Langmuir isotherm parameters can be expressed by a dimensionless constant called the separation factor (*R_L_*), which can indicate the relationship between the adsorbate and the adsorbent, expressed by Equation (5)

(5) $$R_{L}\;=\;\frac{1}{1+K_{L}C_{0}}$$

*R_L_* indicates that the adsorption is unfavorable (*R_L_* > 1), favorable (0 < *R_L_* < 1), linear (*R_L_* = 1) or irreversible (*R_L_* = 0) [40]. The obtained *R_L_* value between 1.11 × 10^−2^ and 2.20 × 10^−2^ varied from the concentration of 300 mg L^−1^ to 600 mg L^−1^, suggesting a favorable process of the adsorption of MB onto BSNC-800-4. Interestingly, *R_L_* decreased with increase of initial concentration of MB, indicating that the adsorption process went more smoothly at high concentration. 

### 2.4. Adsorption Kinetics

Two kinetic models including the pseudo-first-order [41] and the pseudo-second-order [42] were chosen to analyze adsorption mechanism. 

A pseudo-first-order model is expressed as:

(6) $$\log\left(q_{e}-q_{t}\right)\;=\;\log q_{e}-\frac{k_{1}}{2.303}t$$

A pseudo-second-order model is expressed as:

(7) $$\frac{t}{q_{t}}\;=\;\frac{1}{k_{2}q_{e}^{2}}+\frac{1}{q_{e}}t$$

where *k*_1_ represents the pseudo-first-order rate constant and is obtained from the plot of log (*q_e_* − *q_t_*) vs. *t* (min^−1^), *k*_2_ is pseudo-second-order rate constant and is obtained from plot of *t/q_t_* vs. *t* (g mg^−1^ min^−1^).

**Figure 9:**
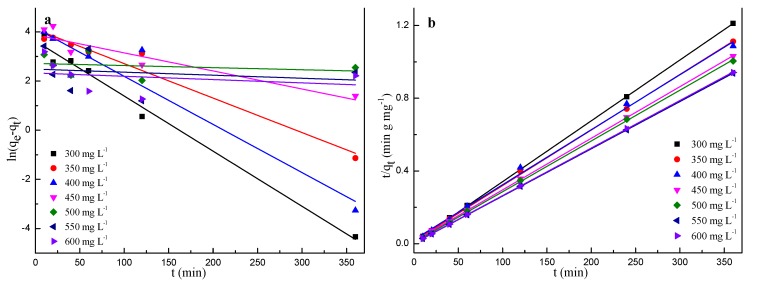
Plots of (**a**) pseudo-first-order, and (**b**) pseudo-second-order for the adsorption of MB onto BSNC-800-4 at different initial MB concentrations at 298 K.

Adsorption kinetic models can give insight on the rate control or adjustment during the adsorption process, which has an influence on the mass transfer and the adsorption time. The two pseudo kinetic models (Figure 9) were investigated and the related parameters were shown in Table 3. It was found that correlation coefficients from the pseudo-second-order model were higher than that of the pseudo-first-order model for all of the MB concentrations. This indicated that the calculated adsorption capacity at equilibrium from pseudo-second-order model would performed better, which is consistent with other studies [43,44]. It was also confirmed that the chemisorption is the main rate controlled step during the adsorption process of BSNC-800-4 and the valence forces through electrons sharing involved between the hydrophilic edges sites of BSNC-800-4 and the MB solution. 

### 2.5. Diffusion Model

The intra-particle diffusion model [45] was chosen to analyze the kinetic data and is expressed as follows:

(8) $$q_{t}\;=\;k_{3}t^{\frac{1}{2}}+C,$$

where *k*_3_ (mg g^−1^ min^−0.5^) is the intra-particle diffusion rate constant. *C* is the intercept. The kinetic parameters were calculated according to the slope and intercept of each liner plot.

In addition, to understand diffusion types during the adsorption process, the kinetic data were also analyzed by Boyd model [46]:

(9) $$B_{t}\;=\;-0.4997-\ln\left(1-\frac{q_{t}}{q_{e}}\right)$$

where *B_t_* is the mathematical function of *q_t_/q_e_*.

**Figure 10:**
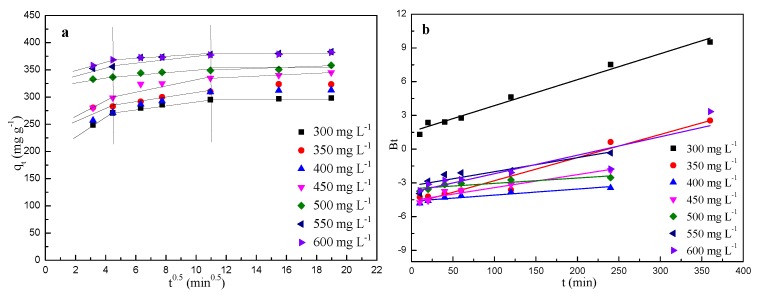
Plots of (**a**) intra-particle diffusion model and (**b**) Boyd model for the adsorption of MB on BSNC-800-4 at 298 K.

The plots of *q_t_* vs. *t*^0.5^ for the MB concentrations indicate three stages (Figure 10a). The first phase with contact time of 10–20 min was the instantaneous adsorption or external surface adsorption, indicating the mass transfer of adsorbate molecules from the bulk solution (MB) to the surface of BSNC-800-4, due to the functional groups and the hierarchical porous structures on the surface of BSNC-800-4. The second stage with contact time of 20–120 min was the gradual adsorption process, indicating that the intra-particle diffusion model was the rate controlling step. The last region was the equilibrium stage, due to the number of adsorption sites and the low concentration of MB solution. It is obvious that the extended curves of Figure 10a did not pass through the origin, and this indicated that intra-particle diffusion was not the only rate-limiting mechanism in the adsorption process. According to the theory of Boyd’s model, if the plot of *B_t_* vs. *t* passes through the origin, the rate controlling step of the adsorption process belongs to pore diffusion; otherwise, it belongs to film diffusion. Our results (Figure 10b) showed the plots did not pass through the origin, indicating that the film diffusion was the rate controlling step between the adsorption of MB onto BSNC-800-4.

## 3. Materials and Methods

### 3.1. Materials

The raw material (bamboo shoot, *Phyllostachys pubescens*) used in this study [47], was collected from Lishui, Zhejiang Province, China. The scale-like outer skin of bamboo shoots were removed, cut into slices and dried at 70 °C for 12.0 h in the oven. Then it was milled and screened to 250–425 um particles. The particles of bamboo shoots were dried at 70 °C in the oven until the mass was stabilized. KHCO_3_ was purchased from Sinopharm Chemicals Co., Ltd (Shanghai, China). MB was purchased from Tianjin Kemiou Chemical Reagent Co., Ltd (Tianjin, China). The chemical formula of MB is C_16_H_18_ClN_3_S (MW = 319.87 g mol^−1^). 

### 3.2. Preparation and Characterization of BSNCs

Two g particles of bamboo shoot were put into KHCO_3_ solution with the mass ratio of bamboo shoot particles and KHCO_3_ of 1:1, 1:2 and 1:4. The mixtures were firstly ultrasound-treated and were freeze-dried until the mass stabilized. They were calcined in a tubular furnace at 600 °C, 700 °C and 800 °C for 1.0 h under nitrogen atmosphere with the heating rate of 10 °C min^−1^. After cooling down to ambient temperature, then rinsed using deionized water until the pH of the washing solution reached to 7. Then the samples were dried in the oven at temperature of 70 °C until the mass was stabilized. They were labeled as BSNC-T-R, where “T” and “R” represents the calcination temperature and the mass ratio of KHCO_3_ to bamboo shoot particles. The sample without adding chemical activating agent and calcined at 800 °C under N_2_ atmosphere was labeled as BS-800. 

The C, H and N content of the BSNCs were determined by an elemental analyzer (Vario EL IIICHNS; Elementar, Langenselbold, Germany). The morphology of the carbons was examined using the field emission scanning electron microscope (SEM) (FEI Quanta 200 HV; Waltham, MA, USA). The surface chemical components of the BSNCs were tested on an X-ray Photoelectron Spectroscopy (XPS) (Thermo Scientific Escalab 250Xi; Thermo Fisher Scientific, Waltham, MA, USA) using a Vacuum Generators XPS system operating with Al (K_α_) radiation. N_2_ adsorption-desorption was determined on a sorption analyzer (Quantachrome Autosorb 2020; Quantachrome, Boynton, FL, USA). The samples were degassed at temperature of 180 °C for 6.0 h and analyzed at temperature of 77 K. The specific surface area, pore volume and pore size distribution of the samples were calculated by the Brunauer–Emmett–Teller (BET) method, t-plot method and non-local density functional theory (NLDFT).

### 3.3. Adsorption Equilibrium

The adsorption capacities of BSNCs for MB were investigated in batch mode. The stock solution of MB was 1000 mg L^−1^, which was prepared by dissolving 1 g of MB in 1 L deionized water. Different initial concentrations of MB were obtained through diluting the stock solution. The volume of MB solution was 50 mL and the given amount of the BSNCs was added to the MB solutions for each experiment. The conical flasks were fixed on a shaker with the speed of 200 rpm at 298 K. The remaining concentration of MB was determined at different time intervals of 10 min, 20 min, 30 min, 60 min, 120 min, 240 min and 360 min by UV-vis spectrometer (PerkinElmer, Waltham, MA, USA) at 665 nm. Three replicates of each experiment were performed.

The adsorbed amount of MB per unit mass of the adsorbent (*q_e_*, mg g^−1^) and the removal efficiency (*R*) of MB at equilibrium were calculated based on Equations (10) and (11).

(10) $$q_{e}\;=\;\frac{(C_{0}-C_{e})V}{m}$$

(11) $$Removal\left(\%\right)=\;\frac{C_{0}-C_{e}}{C_{0}}\;\times \;100$$

where *C*_0_ and *C_e_* are the initial and equilibrium concentrations of MB (mg L^−1^), respectively. *m* is the mass of the adsorbent used (g), *V* is the volume of MB solution (L). 

### 3.4. Adsorption Kinetic

The adsorption kinetics were derived from the data at different time intervals and the concentrations of MB were calculated. The amount adsorption at time *t*, *q_t_* (mg g^−1^) was calculated by Equation (12):

(12) $$q_{t}\;=\;\frac{(C_{0}-C_{t})V}{m}$$

where *C*_0_ and *C_t_* are the concentration of MB at initial and time *t* (mg L^−1^), respectively. m is the mass of the adsorbent used (g), *V* is the volume of MB solution (L). 

## 4. Conclusions

This study has clearly revealed that bamboo shoot—a widespread and easily available raw material—was found to be a superb precursor to prepare porous nitrogen self-doped activated carbons. A carbon for adsorption of MB from bamboo shoot (BSNC-800-4) can be optimally achieved at the temperature of 800 °C and the mass ratio of bamboo shoot particles to KHCO_3_ of 1:4. This carbon material (BSNC-800-4) performs excellent in adsorption capacity and removal efficiency of MB. The adsorption process onto MB corresponds to monolayer adsorption and is controlled by film diffusion. The pseudo-second-order model could accurately estimate the adsorption capacity of the carbon material at an equilibrium state.

## Figures and Tables

**Table 1:** Porosity properties and chemical composition of BSNCs.

Samples	S_BET_ (m^2^ g^−1^)	V_T_ (m^3^ g^−1^)	Vµ (m^3^ g^−1^)	D_p_ (nm)	N (wt%)	H (wt%)	C (wt%)	O (wt%)
BS-800	12.2	-	-	-	4.64	1.05	63.87	28.72
BSNC-700-1	1208.2	-	-	1.97	2.71	0.91	67.27	28.83
BSNC-700-2	1215.1	0.59	0.52	1.94	2.63	0.87	84.12	12.19
BSNC-600-4	962.8	0.48	0.34	1.97	4.65	1.96	69.18	23.97
BSNC-700-4	1475.5	0.73	0.63	1.97	2.79	0.90	80.37	15.78
BSNC-800-4	2270.9	1.25	0.94	2.19	1.06	0.80	43.43	54.36

**Table 2:** Isotherm parameters for the adsorption of MB onto BSNC-800-4 at 298 K.

**Isotherms**	**Parameters**		
Langmuir	*Q*_0_ (mg g^−1^)	*K*_L_ (L mg^−1^)	*R* ^2^
	384.6	0.148	0.9962
Freundlich	*K_F_* ((mg g^−1^) (L mg^−1^)^1/n^)	n	*R* ^2^
	261.2	1.50	0.9043
Temkin	*K_T_*	B	*R* ^2^
	11.26	22.4	0.8808

**Table 3:** Kinetic model parameters for the adsorption of MB onto BSNC-800-4 at different initial MB concentrations at 298 K.

*C*_0_ (mg g^−1^)	*q_e,exp_* (mg g^−1^)	Pseudo-First-Order Kinetic Model			Pseudo-Second-Order Kinetic Model		
		*k*_1_ (min^−1^)	*q_e,cal_* (mg g^−1^)	*R* ^2^	*k*_2_ (g mg^−1^ min^−1^)	*q_e_* (mg g^−1^)	*R* ^2^
300	297.0	0.0537	37.7	0.98	0.0016	303.0	0.99
350	323.8	0.1935	60.1	0.76	0.0007	322.6	0.99
400	331.2	0.0101	57.2	0.59	0.0004	333.3	0.99
450	349.2	0.0168	48.5	0.87	0.0006	357.1	0.99
500	358.3	0.0481	51.6	0.71	0.0012	357.1	0.99
550	384.1	0.0288	15.8	0.84	0.0029	384.6	0.99
600	382.2	0.0378	27.8	0.84	0.0022	384.6	0.99

## References

[1] Vandevivere P.C., Bianchi R., Verstraete W. (1998). Review: Treatment and reuse of wastewater from the textile wet-processing industry: Review of emerging technologies. Cheminform.

[2] Pathania D., Sharma S., Singh P. (2017). Removal of methylene blue by adsorption onto activated carbon developed from Ficus carica, bast. Arabian J. Chem..

[3] Albadarin A.B., Collins M.N., Mu N., Shirazian S., Walker G., Mangwandi C. (2017). Activated lignin-chitosan extruded blends for efficient adsorption of methylene blue. Chem. Eng. J..

[4] Worch E. (2012). Adsorption Technology in Water Treatment.

[5] Bratby J. (2006). Coagulation and flocculation in water and wastewater treatment-second edition. Drink. Water Treat..

[6] Deng Y., Zhao R. (2015). Advanced oxidation processes (AOPs) in wastewater treatment. Curr. Poll. Rep..

[7] Jiao S., Zhao Y., Bi M., Bi S., Li X., Wang B., Li C., Dong Y. (2018). Removal of methylene blue from water by BiFeO_3_/carbon fibre nanocomposite and its photocatalytic regeneration. Catalysts.

[8] Chen M., Jafvert C. (2018). Application of cross-linked stearic acid nanoparticles with dialysis membranes for methylene blue recovery. Sep. Purif. Technol..

[9] Eldin M.S.M., Gouda M.H., Abu-Saied M.A., El-Shazly Y.M.S., Farag H.A. (2015). Development of grafted cotton fabrics ions exchanger for dye removal applications: Methylene blue model. Desalin. Water Treat..

[10] Hameed B.H., Din A.T.M., Ahmad A.L. (2007). Adsorption of methylene blue onto bamboo-based activated carbon: Kinetics and equilibrium studies. J. Hazard. Mater..

[11] Hameed B.H., EI-Khaiary M.I. (2008). Equilibrium, kinetics and mechanism of malachite green adsorption on activated carbon prepared from bamboo by K_2_CO_3_ activation and subsequent gasification with CO_2_. J. Hazard. Mater..

[12] Borah L., Goswami M., Phukan P. (2015). Adsorption of methylene blue and eosin yellow using porous carbon prepared from tea waste: Adsorption equilibrium, kinetics and thermodynamics study. J. Environ. Chem. Eng..

[13] Zhou L., Yu Q.Y., Cui Y., Xie F., Li W.J., Li Y.W., Chen M.F. (2017). Adsorption properties of activated carbon from reed with a high adsorption capacity. Ecol. Eng..

[14] Ahmed M.J., Okoye P.U., Hummadi E.H., Hameed B.H. (2019). High-performance porous biochar from the pyrolysis of natural and renewable seaweed (*Gelidiella acerosa*) and its application for the adsorption of methylene blue. Bioresour. Technol..

[15] Liu R.L., Liu Y., Zhou X.Y., Zhang Z.Q., Zhang J., Dang F.Q. (2014). Biomass-derived highly porous functional carbon fabricated by using a free-standing template for efficient removal of methylene blue. Bioresour. Technol..

[16] Piccin J.S., Gomes C.S., Feris L.A., Gutterres M. (2012). Kinetics and isotherms of leather dye adsorption by tannery solid waste. Chem. Eng. J..

[17] Maneerung T., Liew J., Dai Y., Kawi S., Chong C., Wang C.H. (2016). Activated carbon derived from carbon residue from biomass gasification and its application for dye adsorption: Kinetics, isotherms and thermodynamic studies. Bioresour. Technol..

[18] Liu Z., Xiao K., Guo H., Ning X., Hu A., Tang Q., Fan B., Zhu Y., Chen X. (2017). Nitrogen-doped worm-like graphitized hierarchical porous carbon designed for enhancing area-normalized capacitance of electrical double layer supercapacitors. Carbon.

[19] Ma Z., Zhang H., Yang Z., Ji G., Yu B., Liu X., Liu Z. (2016). Mesoporous nitrogen-doped carbons with high nitrogen contents and ultrahigh surface areas: Synthesis and applications in catalysis. Green Chem..

[20] Tan J.Z.Y., Nursam N.M., Xia F., Sani M.A., Li W., Wang X.D., Caruso R.A. (2017). High-performance coral reef-like carbon nitrides: Synthesis and application in photocatalysis and heavy metal ion adsorption. ACS Appl. Mater. Interfaces.

[21] Zhou X., Wang P.L., Zhang Y.G., Wang L.L., Zhang L.T., Zhang L., Xu L., Liu L. (2017). Biomass based nitrogen-doped structure-tunable versatile porous carbon materials. J. Mater. Chem. A.

[22] Sun J., Ding Z.Q., Gao Q., Xun H., Tang F., Xia E.D. (2016). Major chemical constituents of bamboo shoots (*Phyllostachys pubescens*): Qualitative and quantitative research. J. Agric. Food Chem..

[23] Moosa A.A., Ridha A.M., Kadhi N.A. (2016). Use of biocomposite adsorbents for the removal of methylene blue dye from aqueous solution. Am. J. Mater. Sci..

[24] Chen L., Li Y., Du Q., Wang Z., Xia Y., Yedinak E., Lou J., Ci L. (2017). High performance agar/graphene oxide composite aerogel for methylene blue removal. Carbohydr. Polym..

[25] Wang C., Zhou J., Chu L. (2015). Chlorine-functionalized reduced graphene oxide for methylene blue removal. RSC Adv..

[26] Kharismadewi D., Haldorai Y., Nguyen V.H., Tuma D., Shim J.J. (2016). Synthesis of graphene oxide-poly (2-hydroxyethyl methacrylate) composite by dispersion polymerization in supercritical CO_2_: Adsorption behavior for the removal of organic dye. Compos. Interface.

[27] Pels J.R., Kapteijn F., Moulijn J.A., Zhu Q., Thomas K.M. (1995). Evolution of nitrogen functionalities in carbonaceous materials during pyrolysis. Carbon.

[28] Sevilla M., Parra J.B., Fuertes A.B. (2013). Assessment of the role of micropore size and N-doping in CO_2_ capture by porous carbons. ACS Appl. Mater. Interfaces.

[29] Wang W.Y., Zhu J.J., Wang T., Zhao Y.X., Zhao Z. (2016). Mesoporous graphitic carbon nitride: A new and efficient adsorbent for adsorption removal of methylene blue. Sci. Sin. Chim..

[30] Hameed B.H. (2009). Evaluation of papaya seeds as a novel non-conventional low-cost adsorbent for removal of methylene blue. J. Hazard. Mater..

[31] Oei B.C., Ibrahim S., Wang S., Ang H.M. (2009). Surfactant modified barley straw for removal of acid and reactive dyes from aqueous solution. Bioresour. Technol..

[32] Hameed B.H., Rahman A.A. (2008). Removal of phenol from aqueous solutions by adsorption onto activated carbon prepared from biomass material. J. Hazard. Mater..

[33] Langmuir I. (1918). The adsorption of gases on plane surfaces of glass, mica and platinum. J. Chem. Phys..

[34] Freundlich H.M. (1906). Over the adsorption in solution. J. Phys. Chem. A.

[35] Temkin M.J., Pyzhev V. (1960). Recent modifications to Langmuir isotherms. Acta Physic. USSR.

[36] Bao C., Ma J., Zhou L., Shao Y., Wu Q., Fei W. (2015). Self-template synthesis of hierarchical magnetic porous carbon fibers derived from Fe(BTC)-coated bamboo fibers for fast removal of methylene blue. RSC Adv..

[37] Feng Y.F., Zhou H., Liu G.H., Qiao J., Wang J.H., Lu H.Y., Yang L.Z., Wu Y.H. (2012). Methylene blue adsorption onto swede rape straw (*Brassica napus* L.) modified by tartaric acid: Equilibrium, kinetic and adsorption mechanisms. Bioresour. Technol..

[38] Ünal G., Kocabıyık B., Üner O. (2015). Adsorptive removal of methylene blue from aqueous solution by the activated carbon obtained from the fruit of catalpa bignonioides. Water Air Soil Poll..

[39] Jia Z.G., Li Z.Y., Ni T., Li S. (2017). Adsorption of low-cost absorption materials based on biomass (Cortaderia selloana flower spikes) for dye removal: Kinetics, isotherms and thermodynamic studies. J. Mol. Liq..

[40] Vargas A.M.M., Cazetta A.L., Kunita M.H., Silva T.L., Almeida V.C. (2011). Adsorption of methylene blue on activated carbon produced from flamboyant pods (Delonix regia): Study of adsorption isotherms and kinetic models. Chem. Eng. J..

[41] Lagergren S. (1898). Zur theorie der sogenannten adsorption geloester stoffe. Kungliga Sven. Vetenskapsakad Handl..

[42] Ho Y.S., Mckay G. (1999). Pseudo-second order model for sorption processes. Process Biochem..

[43] Wang S., Xu S., Liu C., Chen F., Wang D., Liu S., Chen Z., Wu Z. (2016). Characterization and adsorption behaviors of a novel synthesized mesoporous silica coated carbon composite. Chin. J. Chem. Eng..

[44] Gao S., Liu L., Tang Y., Jia D., Zhao Z., Wang Y. (2016). Coal based magnetic activated carbon as a high performance adsorbent for methylene blue. J. Porous Mat..

[45] Weber W.J., Morris J.C. (1963). Kinetics of adsorption on carbon from solution. J. Sani. Eng. Division.

[46] Boyd G.E., Myers L.S., Adamson A.W. (1947). The exchange adsorption of ions from aqueous solutions by organic zeolites. III. performance of deep adsorbent beds under non-equilibrium conditions. JACS.

[47] Mi B.B., Chen X.F., Jiang C.L., Wang J.X., Chen X.J., Zhang B., Liu X.M., Liu Z.J., Fei B.H. (2018). Nitrogen-doped porous carbon derived from bamboo shoot as solid base catalyst for Knoevenagel condensation and transesterification reactions. Catalysts.

